# High frequency of antimicrobial resistance in *Salmonella* and *Escherichia coli* causing diarrheal diseases at the Yirimadio community health facility, Mali

**DOI:** 10.1186/s12866-024-03198-4

**Published:** 2024-01-23

**Authors:** Bintou Diarra, Ibréhima Guindo, Boī Koné, Maīmouna Dembélé, Ibrahim Cissé, Souleymane Thiam, Kadidia Konaté, Mamadou Tékété, Almoustapha Maīga, Oumou Maīga, Lassina Timbiné, Abdoulaye Djimde

**Affiliations:** 1 Pathogens genomic Diversity Network Africa (PDNA), Sotuba, Bamako, Mali; 2 African Association for research and control of Antimicrobial Resistance (AAAMR), Koulouba, Bamako, Mali; 3 Malaria Research and Training Center - University of Science, Techniques and Technologies of Bamako, Bamako, Mali; 4 National Institute for Public Health Research (INSP), Bamako, Mali; 5 Charles Mérieux Infectiology Center (CICM), Bamako, Mali; 6 CHU–Gabriel Touré, Bamako, Mali; 7 Kumasi Centre for Collaborative Research in Tropical Medicine, BNITM, Kumasi, Ghana; 8 Yirimadio Community Health Center, Bamako, Mali

**Keywords:** Diarrhoea, *E. Coli*, ESBL, *Salmonella*, Community Health

## Abstract

**Background:**

Diarrhoea is a public health problem, especially in developing countries where it is the second leading cause of child mortality. In Low Income Countries like in Mali, self-medication and inappropriate use of antibiotics due to the scarcity of complementary diagnostic systems can lead to the development of multidrug-resistant bacteria causing diarrhoea. The objective of this work was to determine the microorganisms responsible for diarrhoea in children under 15 years of age and to characterize their sensitivity to a panel of antibiotics used in a peri-urban community in Mali. The study involved outpatient children visiting the Yirimadio Community Health Centre and diagnosed with diarrhoea. Stool samples from those patients were collected and analysed by conventional stools culture and the susceptibility to antibiotics of detected bacteria was determined by the disc diffusion method in an agar medium.

**Result:**

Overall, 554 patients were included. Children under the age of 3 years accounted for 88.8% (492 of 554) of our study population. Two bacterial species were isolated in this study, *Escherichia coli* 31.8% (176 of 554) and *Salmonella* 2.9% (16 of 554). In the 176, *E. coli* strains resistance to amoxicillin and to cotrimoxazole was seen in 93.8% (165 of 176) and 92.6% ( 163 of 176), respectively. The ESBL resistance phenotype accounted for 39,8% (70 of 176) of *E. coli*. Sixteen (16) strains of *Salmonella* were found, of which one strain (6.3%) was resistant to amoxicillin and to amoxicillin + clavulanic acid. Another one was resistant to chloramphenicol (6.3%). Two strains of *Salmonella* were resistant to cotrimoxazole (12.5%) and two others were resistant to cefoxitin (12.5%).

**Conclusions:**

The data suggest that *E. coli* is frequently involved in diarrhoea in children under 3 years of age in this peri-urban setting of Bamako, Mali, with a high rate of resistance to amoxicillin and cotrimoxazole, the most widely used antibiotics in the management of diarrhoea in this setting.

## Background

Diarrhoea is a public health problem where sub-Saharan Africa and South Asia bear the highest burden of the disease [1]. It is the leading cause of death worldwide, especially among infants and young children [2], with 1.57 million deaths recorded in 2017 in sub-Saharan Africa [1, 3, 4] .

Most cases of diarrhoea are associated with either the consumption of contaminated water and food or poor sanitation that creates an ideal environment for the easy transmission of diarrheal pathogens. Although the associated mortality has been sharply reduced in many developing countries, mainly due to improvements in general hygiene and advances in health care [5], diarrhoea remains an important cause of hospitalization and death among children [1].

The etiology of diarrhoea varies from one geographical region to another. Pathogens generally associated with bacterial diarrhea include *Salmonella*, *Shigella*, *Yersinia*, *Escherichia coli (E. coli)*, *Campylobacter*, *Vibrio*, with an emergence of *E. coli* in infants [1]. Other causes of diarrhoea are parasites or viruses. *E. coli* is considered a normal colonizer of the digestive microflora, but it also causes various diarrhea and colitis regularly acquired by food-borne infections and production of toxin. Lethality varies from 3 to 5% and more than a third of patients have long-term renal sequelae for entero-hemorrhagic strains [6].

In Mali, diarrhea is the 3rd reason for outpatient visits of children under 5 years of age. A national survey showed that nearly one in ten children had a diarrheal episode in the preceding 2 weeks. The prevalence among infants aged 6–11 months and 12–23 months was 12.8% and 13%, respectively. These ages of high prevalence correspond to the ages at which children begin to receive food supplements and explore their environment, which puts them at greater risk of contamination by pathogens [6].

Treatment is based on the replacement of fluids and electrolytes but also antibiotic therapy in case of diarrhea of bacterial origin. A resistance of 86.4% to amoxicillin, 64.9% to fluoroquinolone and 21.6% to cefoxitin has been observed in diarrheal children in Bamako [7]. However, most of these cases are treated empirically without knowing the etiological agents or their susceptibility to antimicrobials.

An increase in antimicrobial resistance remains a major concern, hence the urgency of establishing a global antibiotic surveillance system, the results of which can contribute to the adaptation of management and control programs.

This study aimed to identify the Gram-negative Bacilli in stool sample of children aged 0–15 years and to determine their antibiotic susceptibility.

## Method

### Type and location of study

It was a cross-sectional study conducted from December 2021 to March 2023 at the Yirimadio Community Health facility in the suburbs of Bamako, the capital city of Mali. Yirimadio is one of the most populated neighborhoods with 312,722 inhabitants. With over 7,000 outpatient visits per month this Community Health facility is the most important in terms of the number of outpatient visits in the Health District of Commune VI, Bamako, Mali.

### Patients and sampling

This study focused on children from 0 to 15 years of age representing 52% of the population of Mali according to the demographic and health survey of 2018 [8]. We systematically recruited 554 male and female children suffering from diarrhoea in the target ages. Children with Diarrhoea, which was defined as the emission of at least three liquid stools per day were included.

### Sample collection

Stool samples were collected in a sterile container of 20 ml and kept at + 4 °C before being sent to the laboratory for microbiological analyses the same day.

### Macroscopy of stool

The macroscopical aspect of the stools was determined by visual observation and categorized as liquid, soft, mucous and/or bloody.

### Direct examination and gram stain

To assess the flora, we used direct examination and Gram Stain on the stools sample. A stool sample was mixed in 1 ml of physiological water and examined by light microscopy to observe Bacteria shape. A Gram stain was performed to detect the presence of Gram-positive bacteria, Gram-negative bacteria, bacilli, cocci, yeast, and spores. The flora is balanced if the proportion of Gram-positive is between 35 and 45%, Gram-negative between 55 and 65% and yeast around 5%. The flora in unbalanced if one component is significantly high or low. The presence of red blood cells and leukocytes in the suspension was also assessed.

### Isolation and identification of bacteria

**Fig. 1:**
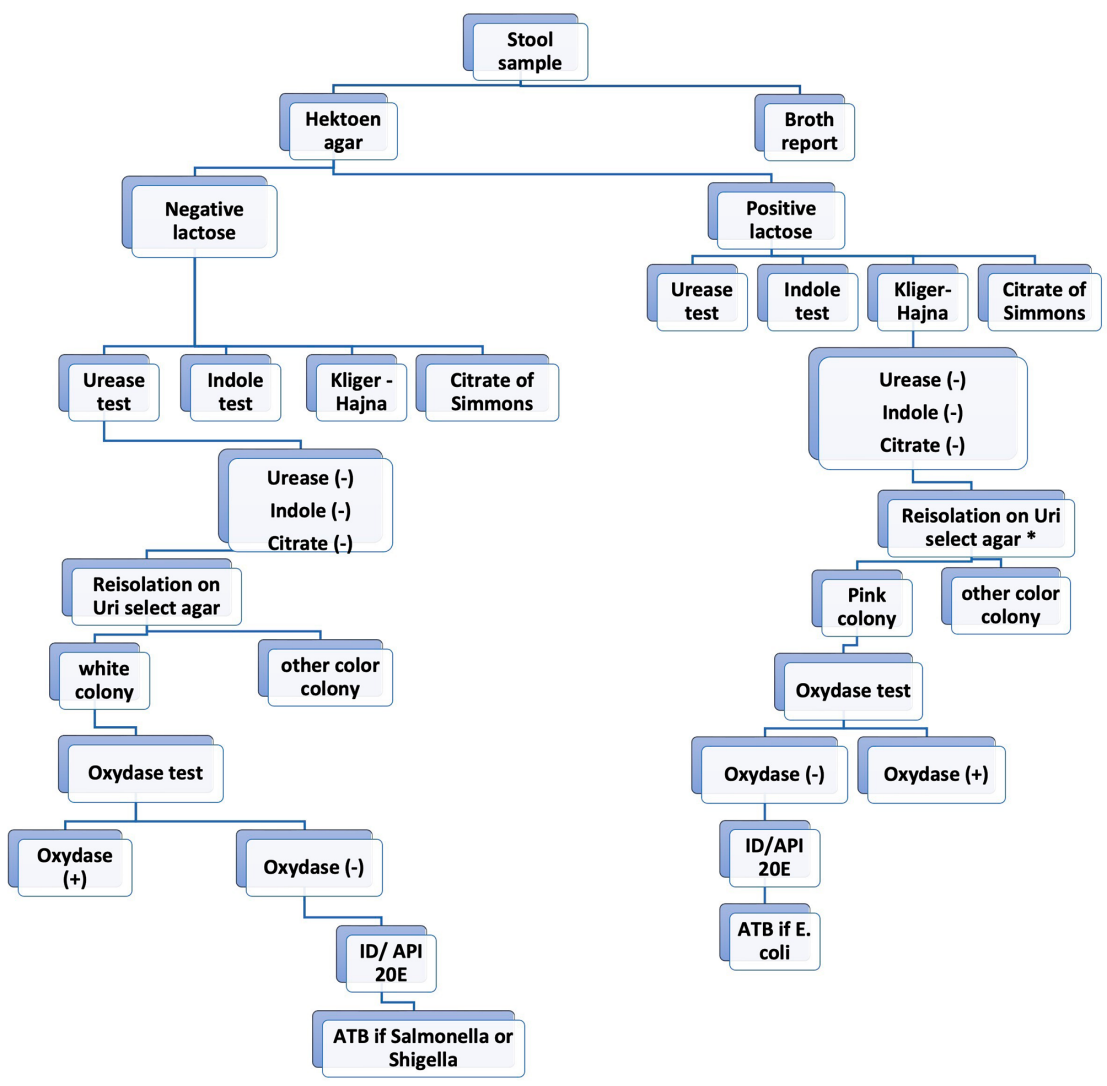
Study flowchart showing laboratory analyses. * if children under 3 years old. **(-)** Negative. **(+)** Positive

Stool suspensions in 1 ml of physiological water were done for each sample. We used a 10 µl calibrated sterile loop to inoculate Hektoen and SS agar using the fractionated inoculation method for all Gram-negative Bacilli research. Half of the suspension volume was used at the same time to systematically inoculate selenite broth or Rapaport broth media to facilitate *Salmonella sp* growing. Four (4) hours later, the previousely inoculated selenite/Rapaport media was used to re-inoculate another Hektoen and SS agar (see Fig. 1). All of the inoculated Hektoen and SS agar were incubated at 35 °C+/-2 °C for 18–24 h.

Hektoen agar is a selective medium for enteropathogenic Gram-negative bacteria. The composition of the medium allows the differentiation of colonies fermenting one of 3 sugars (lactose, saccharose and salicin) and/or producing Hydrogen sulfide (H_2_S). Lactose, saccharose and/or salicin positive colonies appear pink or salmon-red, sometimes surrounded by an area of bile precipitation. Colonies not fermenting these sugars appear blue-green or green. Colonies H_2_S appear with a black center.

The oxidase test was also performed on well-isolated suspect colonies. Briefly, we mixed a drop of the Oxidase reagent (1% aqueous solution of N,N,N,N tetramethyl-paraphenylenediamine; BBL™ Becton Dickinson, Sparks, Maryland) with a suspected colony. In the presence of oxygen with bacteria that produce cytochrome oxidase enzymes a blue-purple color will develop.

For *Salmonella* and *Shigella* identification, between 3 and 5 Green colonies of oxidase negative bacteria were used to make an individual suspension with urea-indole medium. Bacteria possessing urease transform urea into ammonium carbonate resulting in alkalinization which causes a purplish red color in the medium in the presence of phenol red (pH indicator). The production of indole is demonstrated by the addition of Kovacs reagent, which acts with the indole giving a red color in the upper part of the medium in the event of a positive reaction.

*Salmonella* and *Shigella* are urease negative. We continued their identification (urease negative) starting with the classic biochemical gallery Kligler-Hajna and Citrate of Simmons (Biomerieux, Marcy l’Etoile), followed with API^20E^ (BioMerieux, Marcy-L’étoile, France). Common biochemical and cultural characters were considered for orientation. *Salmonella sp.* appear on Hektoen agar as green colonies with or without a black spot at the center and are oxidase negative, Lactose negative, Salicin négative, Lysine Decarboxylase LDC positive, Ornithine Decarboxylase ODC positive, Gas production, urease negative, H_2_S positive or not.

*Shigella* appear on Hektoen agar as green and transparent colonies and are oxidase negative, Lactose negative, Salicin negative, Citrate of Simmons negative, Lysine Decarboxylase LDC negative, Ornithine Decarboxylase ODC negative, none-production of Gas, urease negative, H_2_S negative, none-motile.

For children under 2 years of age, *E. coli* was systematically researched. Pink oxidase negative colonies from Hektoen agar were inoculated in suspension of Urea-Indole medium. For urease negative cases, indole was revealed by Kovacs reagent to select indole positive cases. We subsequently inoculated a classic biochemical gallery Kligler-Hajna, Mannitol-Mobility and Citrate of Simmons (Biomerieux, Marcy l’Etoile). *E. coli* appear as citrate of Simmons negatives and the Kligler-Hajna medium acidification permitted to confirm lactose and glucose positivity of the strain. The identification of *E. coli* by this classic gallery medium considering Lactose positivity, urease negativity, indole positivity, and Citrate of Simmons negativity, was confirmed by API^20E^ (BioMerieux, Marcy-L’étoile, France). (see Fig. 1)

### Antibiotic susceptibility

Antibiotic susceptibility was determined by Kirby-Bauer disc diffusion method on Muller Hilton’s agar. The agars were seeded with a bacterial suspension with a turbidity of 0.5 McFarland. The Antibiotic disks used and their load were described in Table 1. The cultures were incubated at 35 °C for 24 h. The results were interpreted according to the recommendations of the European Committee of antibiotic susceptibility testing (EUCAST, 2020) [9].

**Table 1:** Loads of antibiotics tested by species

E. coli	Salmonella
Amoxicillin 2 µg / Ampicilin 20 µg	Amoxicillin 2 µg / Ampicilin 20 µg
Amoxi + clavulanic acid 20/10 µg	Ceftriaxone 30 µg
Cefalotin 18 µg	Amoxi + clavulanic acid 20/10 µg
Cefoxitin 30 µg	Cefoxitin 30 µg
Ceftriaxone 30 µg	Ciprofloxacin 5 µg
Cefepime 30 µg	Chloramphenicol 30 µg
Imipenem 10 µg	Sulfamethoxazol/trimethoprim 125/75 µg
Aztreonam 30 µg	Gentamycin 10 µg
Gentamycin 10 µg	
Colistine 10 µg	
Ciprofloxacin 5 µg	
Sulfamethoxazol/trimethoprim 125/75 µg	

Extended Spectrum Beta Lactamase (ESBL) was revealed phenotypically by combining amoxicillin-clavulanic acid/ceftriaxone/cefepime discs using the double disc synergy test.

All strains reported as ESBL in results were Cefoxitin susceptible with resistance to a third generation of cephalosporine.

We considered as multidrug resistant each strain with resistance to at least two different families of antibiotics. For quality control of sterility and growth, we used *E. coli* ATCC® 25,922™ and *Escherichia coli* ATCC®35,218™.

### Data analysis

Socio-demographic information was collected for each patient on individual survey sheets. The data was entered in Excel 2016 and analyzed by SPSS (version 25).

## Results

### Description of population

A total of 554 children were included in our study. Ages ranged from 1 month to 15 years with an average of 16.9 months. Male gender was the most represented with 54% (Table 2). Nearly all patients were from Yirimadio (96.9%). Very few came from neighboring locations.

**Table 2:** Description of population

Sociodemographic Variables	Frequency/Value (*N* = 554)
**Sex**	
**Female** **Male**	46 54
**Ages**	
**Median**	16.9
Less than 3 years	88.8
3–5 years	4.2
More than 5 years	7
**Residence**	
Yirimadio Missabougou Banankabougou Niamana Sokorodji Sirakoro Baguinda	96.9 1.4 0.4 0.4 0.5 0.2 0.2

**Table 3:** Description of stools

Variables	Frequency (*N* = 554)
Macroscopy*	
Liquid	91.3
Soft	8.7
Bloody	0.9
Mucus	18.7
**Flora**	
Balanced	39
unbalanced	61
**Red cell/leukocyt**	
Red cell	24.2
Leukocyt	39.5

* More than one modality may occur in an individual

### Description of stools

Overall 91.3% of stool samples collected from the 554 children were liquid while the remaining stools were soft 8.7% (48 of 554). On the other hand 18.8% (104 out of 554) of stool samples had mucus while 0.9% had visible blood (5 of 554). In addition, we found that 61% (338 of 554) of children had unbalanced bacteria flora (Table 3).

### Bacterial identification

Stool culture allowed us to isolate 192 bacteria pathogens representing a global isolation rate of 34.7%. Among the isolated pathogens 176 were *E. coli* representing an isolation rate of 31.8% and 16 were *Salmonella* representing an isolation rate of 2.9%. *E. coli* represented 91.7% of positive cultures (176 of 192) and *Salmonella* 8.3% (16 of 192). *E. coli* was only found in children under 3 years of age (Table 3) while *Salmonella* was also found in older children.

**Table 4:** Distribution by isolated microorganism by age group

Age range	Frequency
***E. coli*** (*N* = 176)	
< 6 months	14.2
6 months − 1 year	44.3
1–3 years	41.5
***Salmonella*** (*N* = 16)	
1–3 years 3–5 years > 5 years	87.5 6.3 6.3

No case of *Shigella* was identified in this study.

### Antibiotic susceptibility

The 192 strains isolated in our study were tested for susceptibility to 12 antibiotics for *E. coli* and 8 antibiotics for *Salmonella* (Table 1).

*E. coli* susceptibility: More than 50% of the 176 *E. coli* strains were found to be resistant to all beta-lactam tested. However, only 4% (7 of 176) were resistant to imipenem and 15.3% (27 of 176) were resistant to cefoxitin. Overall, 93.8% (165 of 176) and 92.6% (163 of 176) of the *E. coli* strains tested were resistant to amoxicillin and cotrimoxazole, respectively. Rate of *E. coli* resistance to ciprofloxacin and gentamicin were 55.7% (98 of 176) and 33% (58 of 176), respectively. (Table 4)

**Table 5:** Antibiotic resistance profile of *E. coli* and *Salmonella*

Antibiotics	E. coli (*N* = 176)	Salmonella (*N* = 16)
Amoxicillin /Ampicillin	93.8	6.3
Amoxicillin + clavulanic acid	83	6.3
Cefalotin	76.1	-
Cefoxitin	15.3	12.5
Ceftriaxone	52.3	0
Cefepime	64.2	-
Imipenem	4	-
Aztreonam	56.8	-
Cotrimoxazole	92.6	12.5
Ciprofloxacin	55.7	0
Gentamicin	33	0
Colistin	14.2	-
Chloramphenicol	-	6.3

For multidrug resistance, 50% (88 of 176) of *E. coli* were simultaneously resistant to amoxicillin, cotrimoxazole and ciprofloxacin, and 25% (44 of 176) had simultaneous resistance to amoxicillin, co-trimoxazole, ciprofloxacin and gentamicin. (Table 5)

**Table 6:** Multidrug resistance observed in *E. coli and Salmonella*

Isolated Bacteria	Cases and Frequency of multidrug resistance
***E. coli*** (*N* = 176)	
Amoxicillin, cotrimoxazole	157 (89.2)
Amoxicillin, cotrimoxazole and ciprofloxacin	88 (50)
Amoxicillin, cotrimoxazole, ciprofloxacin and gentamicin	44 (25)
***Salmonella*** (*N* = 16)	
Amoxicillin, cefoxitin	1 (6.3)

**Fig. 2:**
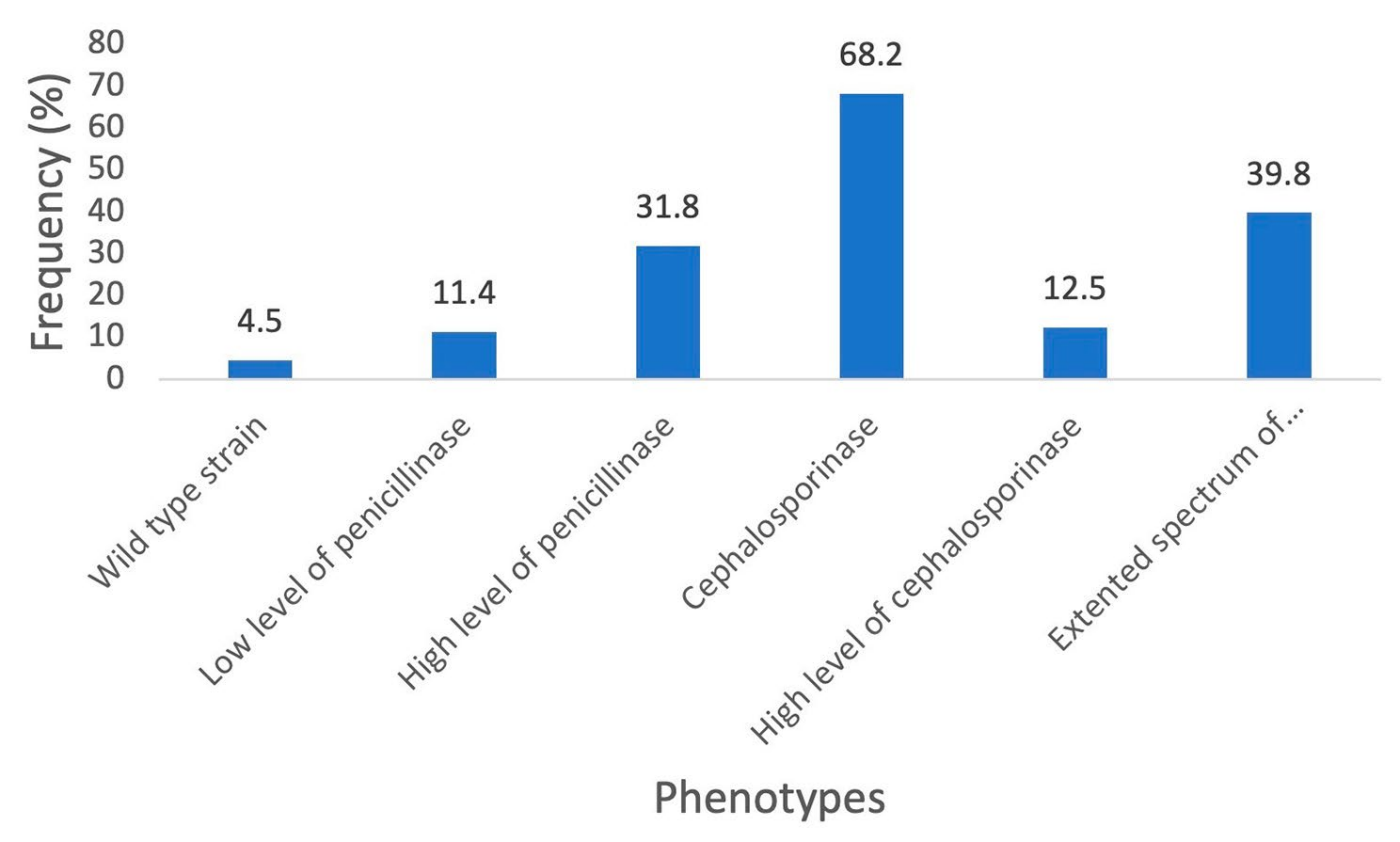
Resistance phenotype of *E. coli*

In the *E. coli* strains (8 of 176) 4.5% had the wild phenotype, 11.4% (20 of 176) had low level of penicillinase, 31.8% (56 of 176) had high level of penicillinase, 68.2% (120 of 176) had cephalosporinase, 12.5% (22 of 176) had high level cephalosporinase and 39.8% (70 of 176) were extended spectrum beta lactamase bacteria (Fig. 2).

*Salmonella* susceptibility: Among the 16 *Salmonella* strains isolated, 5 were found to be resistant to 5 of the 8 antibiotics tested. Indeed, 6.3% (1 of 16) of *Salmonella* were resistant to amoxicillin, amoxicillin-clavulanic acid and chloramphenicol, and 12.5% (2 of 16) were resistance to cotrimoxazole and cefoxitin. Our tested *Salmonella* strains had full susceptibility to ceftriaxone, ciprofloxacin and gentamicin. (Table 4) One strain of *Salmonella* (6.3%) was multidrug resistant i.e. simultaneously resistant to amoxicillin, amoxicillin-clavulanic acid and cefoxitin.

## Discussion

In this cross-sectional study conducted for 15 months with 554 children under the age of 15 years, suffering from diarrhoea and seen in consultation at the community health facility of Yirimadio, Mali *E. coli* showed resistance to several families of antibiotics, including resistance rates of 93.8% to amoxicillin, 92.6% to cotrimoxazole, 55.7% to ciprofloxacin and 33% to gentamicin. These antibiotics are the most prescribed very often empirically. In previous hospital-based studies in Bamako, Amoxicillin represented 30.5% of prescription and 39.8% of antibiotic prescription were done in Pediatric units [10, 11]. This would be the reason for the high rate of resistance to amoxicillin in children and in pediatrics. It is Therefore this high rate of resistance was probably the result of a selection pressure. In addition, self-medication and the unsupervised use of traditional medicines leads to the consumption of substances with antibacterial properties. This excessive consumption of antibiotics, beyond stimulating bacterial resistance, is at the origin of the imbalance of the intestinal flora in this fragile infant population. Beta lactams are one of the most prescribed families, both orally and by injection. The high resistance rates and phenotypes observed are also found in other countries in pediatrics units. In Tchad, a similar rate of resistance to amoxicillin as ours (93.8%) was found in 2017 [12] .

Extended-spectrum beta-lactamase-producing strains have been found in Sudan since 2012 with 32% and in 2018 in China in Tongji where about half of the isolated *E. coli* strains were ESBL-producing [13] .

Gentamicin and ciprofloxacin were among the most prescribed antibiotics in probabilistic antibiotic therapy in hospitals in 2021 in Mali [11]. For fluoroquinolones, the resistance rate was 55,7% in our study against 64.9% previously found in Bamako in a community setting with associated *qnrA*, *qnrB*, *qnrS* genes [7]. As the population was communal in the 2 studies, the difference in prevalence could be due to sampling bias. However, a 21.9% lower ciprofloxacin resistance rate was found in Tchad [12]. Showing a disparity in resistance to fluoroquinolones of strains of *E. coli* isolated from diarrhea.

Gentamicin is a good indicator of resistance for the family of aminoglycosides. This antibiotic is generally prescribed in combination with beta-lactams when signs of severity are observed. Our study showed a resistance rate of 33% against 29.7% previously observed in Bamako in the community. Higher rates of 50% were however found in Tchad [12].

Cases of multidrug resistance have been observed, compromising therapeutic options. The presence of plasmid supports for this multidrug resistance would facilitate the spread of these strains in the community or even hospital settings. In our study, the multidrug resistance rate was 50%. The community study previously carried out in Bamako reported the detection of class 1, 2 and 3 integrons, genetic carriers of multi-resistance, in bacteria [7] showing a real need to strengthen molecular monitoring of resistance. Two meta analyzes presented variable rates of multidrug resistance, notably 66.3% in Asia [14] and 28% in countries with limited resources [15]. A clear trend of multidrug resistance does not emerge, but the risk of evolution toward total resistance remains.

A low resistance rate of *Salmonella* to antibiotics was observed: 6.3% to amoxicillin, amoxicillin + clavulanic acid and chloramphenicol, and 12.5% to cotrimoxazole and cefoxitin. Higher rate resistance (100%) to amoxicillin and cotrimoxazole were observed in Zambia in 2017 [1] .The hospital origin of these strains could explain this difference. However, sensitivity to ciprofloxacin and cephalosporin remains preserved with less than 10% resistance as described in Lomé [16].

Despite high resistance rates to several families of antibiotics, retained sensitivity was observed for antibiotics such as imipenem, cefoxitin and colistin against *E. coli* [12, 16, 17].

Our study population was mainly made up of children under 3 years old, and the male gender was slightly higher (54%) than the female gender. In Bamako during a study on the Pathogenicity Factors and Antibiotic Resistance of *Escherichia coli* strains isolated in Diarrheal Children from 0 to 59 Months at the Community level, a high frequency of male gender (64.2%) was reported in 2022 [7]. Although our study focused on cases of diarrhea in the population aged between 0 and 15 years, children under 3 years old were most represented with 88.8%. This trend is similar to that reported nationally with a high frequency of diarrhea in children under 2 years [18]. This could be explained by the infantile reflexes to carry the hand in the mouth and the introduction of foods other than breastfeeding milk in child alimentation. Also, hygiene issues due to the mothers’ traditional practices can be associated [6] .

The conventional stool culture allowed us to isolate pathogenic bacteria in 34.7% of cases including *E. coli* and *Salmonella*, but no cases of *Shigella* were found. This rate is low compared to those described at Shiraz (Iran) which had an isolation rate of 40.6% [19]. The study of Shiraz was done in a hospital context that considered only presence of white cells as characteristic to perform stool culture [19]. Although the white cells were found in 219 cases in our study all stool samples were systematically cultured.

*E. coli* was isolated in this study with a prevalence of 31.8%. A similar prevalence (30.8%) was found a few years earlier during a District-wide study in four community health facilities of Bamako, the Capital city of Mali [7]. Taken together, the data suggest the rate of *E. coli* isolation from diarrhoea at the community level at Bamako has stabilized. Other studies are needed to assess the rate of *E. coli* associated to diarrhoea at the community level outside of Bamako. In addition, we have no data on the exogen or endogen origins of these *E. coli* strains, which would be required to clarify the role of gut flora in these infections.

In Iran, surveillance carried out in a hospital environment over 2 years in Tehran reported a bacterial isolation rate of 4.23% (310/7321) [20]. This prevalence is lower than ours. This difference would be due to the hospital origin of the cases of diarrhea recorded in Tehran against a community origin in our study. *E. coli* represented 146 cases out of 310, that’s almost half isolated only from children less than 10 years [20] .

Alongside *E. coli*, the *Salmonella* genus was also isolated in our study but with a low proportion of 2.9%. In the West African sub-region, a similar proportion of *salmonella* isolation was found in community settings in Guinea Bissau with 2.4% and a lower one in the same study with 1% in Senegal. This study was carried out in the general population and children aged 0–15 represented around 40% [21]. Rates close to those of West Africa were found in East Africa, notably 1.3% in Ambo town [22]. The rate of *salmonella* isolation seems low in West and East Africa.

However, a high rate of *Salmonella* was found in the Middle East. In Iran during a hospital surveillance (including outpatients and inpatients) over 2 years in Tehran, *Salmonella* was isolated in 83/310 cases and 65 cases among children less than 10 years. *Shigella* was isolated in 79/310 cases and 57 cases among children less than 10 years [17].

No case of Shigella was detected in this study. This could be because we only used classic microbiology but no molecular detection in this study. Indeed other authors found very few cases of Shigella by classical microbiology methods [23], confirming that molecular methods were more sensitive in detecting Shigella [24].

## Conclusions

The study confirmed that *E. coli* plays an important role in the etiology of diarrhea in children. Two species namely *E. coli* and *Salmonella* were isolated in this study and the majority of *E. coli* had resistance to usual antibiotics such as amoxicillin and cotrimoxazole. Cases of multidrug resistance have been found with retained sensitivities for certain high-level antibiotics. Molecular surveillance is necessary to better understand the resistance mechanisms and the transmission of these strains.

## Data Availability

The datasets used and analyzed during this study are available from the corresponding author on reasonable request.

## Abbreviations

*E. coli*: *Escherichia coli*
USTTB: University of Sciences, Techniques and Technologies of Bamako
SPSS: Statistical Package for the Social Sciences
INSP: National Public Health Institute
CHU: University hospital center
MRTC: Malaria Research and Training Center
CICM: Charles Mérieux Infectiology Center
ESBL: Extended spectrum of beta-lactamase
API: Analytical profile index
EUCAST: European Committee of antibiotic susceptibility testing
